# Association between Amyloid Burden and Health‐Related Resource Use

**DOI:** 10.1002/alz.095557

**Published:** 2025-01-09

**Authors:** Carolyn W Zhu, Trey Hedden, Gregory A. Elder, Hillel Grossman, Judith A. Neugroschl, Corbett Schimming, Amy Aloysi, Mary Sano

**Affiliations:** ^1^ James J. Peters VA Medical Center, Bronx, NY USA; ^2^ Icahn School of Medicine at Mount Sinai, New York, NY USA

## Abstract

**Background:**

Several disease modifying treatment (DMT) in early AD have shown effectiveness in reducing rate of decline on cognition and function. Earlier analyses using participants who are likely targets for clinical trial and treatment showed treatment effects may have important effects on patients’ work capacity and need for informal care. However, amyloid pathology was not examined. This study explores the relationship between amyloid burden and health‐related resource use.

**Method:**

Participants enrolled in the Mount Sinai Alzheimer’s Disease Research Center with CDR = 0.5 or 1 and clinical diagnosis of MCI or AD. Health‐related resource use was self‐reported by participant and/or study partner using the Resource Use Inventory (RUI). Amyloid images received a clinical read by a neuro‐radiologist who was blinded to patient characteristics used to determine amyloid status. Two‐part or hurdle models were used adjusting for age, sex, race/ethnicity, education, comorbidities, baseline CDR Sum of Boxes (CDR‐SB), and years of follow up.

**Result:**

Sample included 56 participants (28 amyloid positive, 28 negative) who had 1 or more RUI assessments (average follow up = 2.6±1.5 years). At baseline, average age = 73±8, 17±3 years of schooling, 54% female, 66% White, 20% Black, 11% Hispanic. 91% CDR = 0.5, 13% cognitively normal, 63% MCI, 25% AD; MMSE = 26±3, CDR‐SB = 1.8±1.8, comorbidities = 5.5±2.5.

There were no statistically significant differences (at p = 0.05) in participants' characteristics except that proportion of participants who were White was higher in those who were amyloid positive (86%) than negative (46%) (p = 0.002). No Hispanic participant was amyloid positive. Follow up was longer in those who were amyloid negative (3±1.4 years) than amyloid positive (2.2±1.4 years). Baseline RUI items were not statistically significantly different by amyloid positivity.

At baseline, amyloid positivity was associated with lower likelihood of participation in employment/volunteering. Among those who used help, amyloid positivity was associated with higher home health care hours. Over time, likelihood of receiving informal help increased and participation in employment/volunteering decreased. Data did not reveal any interaction between amyloid positivity and time.

**Conclusion:**

Results from this small sample of participants that reflects AD clinical trial populations suggest amyloid burden may be associated with higher health‐related resource use.

